# Genome-wide sparse canonical correlation of gene expression with genotypes

**DOI:** 10.1186/1753-6561-1-s1-s119

**Published:** 2007-12-18

**Authors:** Elena Parkhomenko, David Tritchler, Joseph Beyene

**Affiliations:** 1 Department of Public Health Sciences, University of Toronto, 155 College Street, Toronto, Ontario, M5T 3M7 Canada; 2 Division of Epidemiology and Statistics, Ontario Cancer Institute, 610 University Avenue, Toronto, Ontario, M5G 2M9 Canada; 3 Hospital for Sick Children Research Institute, 123 Edward Street, Toronto, Ontario, M5G 1X8 Canada

## Abstract

There is a growing interest in studying natural variation in human gene expression. Studies mapping genetic determinants of expression profiles are often carried out considering the expression of one gene at a time, an approach that is computationally intensive and may be prone to high false-discovery rate because the number of genes under consideration often exceeds tens of thousands. We present an exploratory method for investigating such data and apply it to the data provided as Problem 1 of Genetic Analysis Workshop 15 (GAW15). In multivariate analysis, canonical correlation analysis is a common way to inspect the relationship between two sets of variables based on their correlation. It determines linear combinations of all variables from each data set such that the correlation between the two linear combinations is maximized. However, due to the large number of genes, linear combinations involving all single-nucleotide polymorphism (SNP) loci and gene expression phenotypes lack biological plausibility and interpretability. We introduce sparse canonical correlation analysis, which examines the relationships of many genetic loci and gene expression phenotypes by providing sparse linear combinations that include only a small subset of loci and gene expression phenotypes. These correlated sets of variables are sufficiently small for biological interpretability and further investigation. Applying this method to the GAW15 Problem 1 data, we identified groups of 41 loci and 150 gene expressions with the highest between-group correlation of 43%.

## Background

Several studies have demonstrated that there is variation in baseline gene expression levels in humans that has a genotypic component [1,2]. Genome-wide analyses mapping genetic determinants of gene expression are carried out for expression of one gene at a time, which may be prone to a high false-discovery rate and computationally intensive because the number of genes under consideration often exceeds tens of thousands. In this paper we present an exploratory multivariate method for initial investigation of such data and apply it to the data provided as Problem 1 of Genetic Analysis Workshop 15 (GAW15). The linkages between the set of all single-nucleotide polymorphism (SNP) loci and the set of all gene expression phenotypes can be characterized by a type of correlation matrix based on the linkage analysis methodologies introduced by Tritchler et al. [3] and Commenges [4]. In multivariate analysis, a common way to inspect the relationship between two sets of variables based on their correlation is canonical correlation analysis, which determines linear combinations of variables for each data set such that the two linear combinations have maximum correlation. However, due to the large number of genes, linear combinations involving all of the genotypes or gene expression phenotypes lack biological plausibility and interpretability and may not able to be generalized. We have developed a new method, sparse canonical correlation analysis (SCCA), which examines the relationships between many genetic loci and gene expression phenotypes. SCCA provides sparse linear combinations. That is, only small subsets of the loci and the gene expression phenotypes have non-zero loadings so the solution provides correlated sets of variables that are sufficiently small for biological interpretation and further investigation. The method can help generate new hypotheses and guide further investigation.

## Materials and methods

### Data

The data consist of microarray gene expression measurements that are treated as quantitative traits and a large number of genotypes for 14 Centre d'Etude du Polymorphisme Humain (CEPH) families from Utah. Each pedigree contains three generations with approximately eight offspring per sibship. There are 194 individuals, 56 of which are founders. Phenotypes are measured by microarray gene expression profiles obtained from lymphoblastoid cells using the Affymetrix Human Genome Focus Arrays. Morley et al. [2] selected 3554 genes among the available 8793 probes on the basis of higher variation among unrelated individuals than between replicate arrays for the same individual. In this paper, we used a pre-processed and normalized data provided for these genes. Additional phenotypic data obtained for CEPH families includes age and gender.

Genotypes are measured by genetic markers provided by The SNP Consortium and are available for 2882 autosomal and X-linked SNPs. The physical map for SNP locations is also available.

### The statistical model

In this study we are interested in identifying linear combinations of measures based on gene expressions and SNP-based measures that have the largest correlation. Canonical correlation analysis (CCA) establishes such relationships between the two types of variables [5]. Suppose that *x *is a random vector of the first type of variables and *y *is a random vector of the second type of variables. We are looking for vectors *a *and *b *which maximize the following correlation:

$$cor(\begin{array}{cc} a^{\prime }x, & b^{\prime }y \end{array})=\rho (\begin{array}{cc} a, & b \end{array})=\frac{a^{\prime }\Sigma_{12}b}{{\left(a^{\prime }\Sigma_{11}ab^{\prime }\Sigma_{22}b\right)}^{1/2}}$$

where Σ_11_, Σ_22_, and Σ_12 _are the variance and covariance matrices. The solution is obtained from the singular value decomposition (SVD) of a matrix *K *given by:

$$K=\Sigma_{11}^{-1/2}\Sigma_{12}\Sigma_{22}^{-1/2}=UDV^{\prime }=(\alpha_{1},\ldots ,\alpha_{k})D(\beta_{1},\ldots ,\beta_{k}{)}^{\prime }.$$

Here *k*, is the rank of *K*. The highest canonical correlation is obtained by considering the best rank 1 approximation to the matrix *K *[6], *K *= *d*_1_*α*_1_*β*_1_', where *d*_1 _is the positive square root of the first eigenvalue of K'K or KK', and *α*_1 _and *β*_1 _are first singular vectors. Then the canonical vectors or weights in the linear combinations of the two sets of variables that have the largest correlation are given by

$$\begin{array}{ccc} a=\Sigma_{11}^{-1/2}\alpha_{1} & \text{and} & b=\Sigma_{22}^{-1/2}\beta_{1}. \end{array}$$

In conventional CCA, all variables are included in the fitted linear combinations. However, in microarray and genome-wide data, the number of genes under consideration often exceeds tens of thousands. In these cases linear combinations of all features may not be easily interpreted. Sparse canonical correlation analysis (SCCA) enhances biological interpretability and provides sets of variables with sparse loadings. This is consistent with the belief that only a small proportion of genes are expressed under a certain set of conditions, and that expressed genes are regulated at a subset of genetic locations. We propose obtaining sparse linear combinations of features by considering a sparse singular value decomposition of *K *where singular vectors *α *and *β *in Eq. (3) have sparse loadings. We developed an iterative algorithm that alternately approximates the left and right singular vectors of the SVD using soft-thresholding for feature selection. This approach is related to the sparse principal component analysis method of Zou et al. [7] and partial least squares methods described by Wegelin [8].

### Sparse canonical correlation analysis algorithm

Assume that variables in both data sets have been standardized to have zero means and unit variances and *K *is the matrix in Eq. (2). Our algorithm works as follows:

1. Select sparseness parameters *λ*_*α *_and *λ*_*β *_for left and right singular vectors, respectively. Select initial values for singular vectors *α*^0 ^and *β*^0^, set *i *= 0.

2. Update left singular vector *α *as follows:

a. Set *α*^*i*+1 ^= *Kβ*^*i *^and normalize it to have unit length

b. Soft-thresholding: $\alpha^{i+1}={\left(\left|\alpha^{i+1}\right|-\frac{1}{2}\lambda_{\alpha }\right)}_{+}Sign(\alpha^{i+1})$

c. Normalize *α*^*i*+1 ^again

3. Update right singular vector *β *as follows:

a. Set *β*^*i*+1 ^= *Kα*^*i*+1 ^and normalize it to have unit length

b. Soft-thresholding: $\beta^{i+1}={\left(\left|\beta^{i+1}\right|-\frac{1}{2}\lambda_{\beta }\right)}_{+}Sign(\beta^{i+1})$

c. Normalize *β*^*i*+1 ^again

*4. i *= *i *+ 1, repeat steps 2 and 3 until convergence.

The algorithm converges to the first singular vectors *α*_1 _and *β*_1 _in Eqs. (2) and (3). Other canonical vectors can be obtained by considering the residual of the correlation matrix. This is a very computationally efficient algorithm because it usually converges in less than ten iterations and requires only matrix multiplication. Optimal combination of sparseness parameters for soft-thresholding steps can be selected using cross-validation. A subset of the data is repeatedly used to identify linear combinations of variables for the specific pair of sparseness parameters and then the correlation between the obtained canonical vectors is calculated in the remainder of the data set. The optimal combination of *λ*_*α *_and *λ*_*β *_corresponds to the highest average correlation over the repetitions.

### Analysis approach

In this study one type of variables is based on gene expression levels and the other type of information relates to SNP genotypes and pedigree structure. An immediate challenge in this context is how to define correlation between these two types of data. We adopted a measure of covariance of genetic similarity with phenotypic similarity as in the linkage analysis methodologies of Tritchler et al. [3] and Commenges [4]. Consider the offspring generation in all available pedigrees and take all possible sib pairs. Let *y*_*ij *_and *y*_*ik *_be the phenotypes for the siblings *j *and *k *in family *i *for a particular gene expression and let *w*_*ijk *_represent identity-by-descent (IBD) value for these siblings for some specific SNP. Then, for the given gene expression and SNP, the test statistic for linkage in Tritchler et al. [3] is

$$\sigma =\sum_{i}\sum_{j}\sum_{k>j}\left\{y_{ij}-E(y_{ij})\right\}\left\{y_{ik}-E(y_{ik})\right\}\left\{w_{ijk}-E(w_{ijk})\right\},$$

which is used for computation of covariance matrix between the phenotypic similarity and genotypic similarity. Note the similarity of the above expression to Haseman-Elston regression. In fact, Tritchler et al. [3] show that the correlation statistic subsumes both the original Haseman-Elston regression analysis and the later Haseman-Elston (revisited).

### Phenotypic similarity

The phenotypes in this study are the gene expression values for siblings in the last generation of the pedigrees (i.e., the offspring generation). Previous studies have shown that there is variation in human gene expression according to age and gender [2]. Therefore, we limit the analysis to the last generation in all pedigrees and also correct for the effects of gender and age by fitting a linear model

***y*_*ij *_**= *α *+ *β*_***gender ***_***gender*_*ij *_**+ *β*_***age***_***age*_*ij *_**+ ***e*_*ij *_**= ***E***(***y*_*ij*_**) + ***e*_*ij*_**.

Gender and age information were not available for all individuals in pedigree 1454 and for three individuals in pedigree 1340. Therefore, these data were excluded from the analysis. In the 13 remaining pedigrees, there were 344 distinct sib pairs with sibship size varying between 15 and 28. Although sib pairs are correlated within pedigrees, this does not affect the results because this is an exploratory study and no assumption of independence is made.

The phenotypic similarity for siblings *j *and *k *in pedigree *i *is computed as sib-pair cross-product of mean corrected gene expression phenotypes:

{*y*_*ij *_- *E*(*y*_*ij*_)}{*y*_*ik *_- *E*(*y*_*ik*_)}.

### Genotypic similarity

For each sib pair, the probabilities of sharing 0, 1, and 2 alleles identically by descent (IBD) were estimated using MERLIN. This is an exploratory analysis of all SNPs and gene expressions, so great precision in the estimation of the IBD values is not required and the provided physical distance map of the SNP locations is a suitable approximation to the genetic distances required by MERLIN. Given the incomplete genetic marker information for some individuals, exact IBD values could not be computed. We estimated the number of alleles shared IBD by two siblings as a posterior expected value based on the probabilities estimated using MERLIN. Expected IBD values *E*(*w*_*ijk*_) were computed as the sample means over all sib pairs.

### Standardization

We standardize the phenotypic and genotypic similarity variables by subtracting the means and dividing by the standard deviations. Simulations show that after data standardization, the analysis can be simplified by replacing Σ_11 _and Σ_22 _in Eq. (2) with an identity matrix while yielding satisfactory results. Then *K *in Eq. (2) is the covariance between the two standardized data sets and the first canonical vectors in Eq. (3) are just *α*_1 _and *β*_1_.

### Evaluation

We evaluated the performance of SCCA using the leave-one-out cross-validation (LOOCV) analysis, treating a pedigree as one unit. In this study, assessment of performance is based on the estimated test sample correlation. It shows correlation between linear combinations of identified loci and gene expressions in the independent sample. We used pedigree as the unit in LOOCV because it represents a statistically independent unit. Leaving out one whole pedigree preserves dependence structure in the family-based study and ensures independence between training and testing samples. We carried out an analogue of 13-fold CV where fold-size was dictated by the complex structure of the data.

## Results

Using cross-validation, we obtained soft-threshold value of 0.07 for gene expressions and 0.13 for SNPs, corresponding to the maximal test sample correlation. We carried out SCCA using this optimal combination of sparseness parameters and identified groups of 41 SNPs and 150 gene expressions with between-group correlation of 43%. All obtained SNPs are uniformly distributed over a region on chromosome 9 between 86.80 megabases (Mb) and 120.09 Mb. Expressed genes selected by SCCA are distributed over different chromosomes. Six of the identified gene expressions are located on chromosome 9. Three other chromosomes that have more than 15 gene expressions each are 1, 2, and 6. No *cis*-acting genetic regulators were found, where *cis*-regulators are defined as those that map within a 5-Mb region, as was previously defined in Morley et al. [2].

Table 1 summarizes the results of the cross-validation study comparing the performance of SCCA to the complete SVD solution that includes all 3554 gene expressions and 2882 SNPs. Average overlap between the group of 150 gene expressions selected using SCCA on the complete data and the groups of gene expressions selected in CV steps is 46 genes; the average intersection of the 41 SNPs with the results in CV steps is 34 SNPs. Inspecting CV iterations shows pedigrees to be heterogeneous, with two pedigrees, 1416 and 1418, being outliers.

**Table 1:** Summary of prediction results for SCCA and full SVD averaged over 13 leave-one-out-cross-validations

	Number of gene expressions selected	Number of SNPs selected	Average test sample correlation of canonical vectors
SCCA	83	66	0.1144
SVD	3554	2882	0.1384

## Discussion

In this study we presented sparse canonical correlation analysis and demonstrated the application of this new multivariate method to the simultaneous analysis of gene expression levels and SNPs. Due to complex interaction between genes, a set of several loci may be associated with several gene expressions possibly belonging to the same regulatory pathway or genetic network. SCCA discovers such sets of SNP loci and gene expression phenotypes while keeping the size of groups sufficiently small for biological interpretation.

We identified a specific region on chromosome 9 that regulates a group of gene expression profiles. Selected sets of loci should be interpreted as whole in relation to the whole set of selected gene expressions. This sparse solution may help to generate new hypotheses and isolate groups of loci and gene expressions for future biological experimentation. For instance, selection of a specific region on chromosome 9 by SCCA is particularly interesting, and a possible interpretation could be that we found a regulatory region.

The same region on chromosome 9 was also identified in other GAW15 contributions. For instance, considering a small set of genes associated with the development of the enteric nervous system (ENS), Lantieri et al. [9] also found evidence of linkage for two genes, 201387_s_at and 209034_at, to a unique common regulator located on chromosome 9 at 109 centimorgans (cM). Similarly, Wang et al. [10] found 10 gene expressions mapped to a "hot spot" on chromosome 9, however, gene names and specific chromosomal locations were not provided. One of the 41 SNPs selected by SCCA, rs1355620, was found to be linked to a composite score for "cell surface receptor linked to signal transduction" by Liu et al. [11].

We used leave-one-out cross-validation to evaluate the performance of SCCA method. The results showed a slightly lower average test sample correlation for SCCA compared to full SVD solution as shown in Table 1. For this particular data set, a possible explanation is that outliers among the results in the CV steps due to the two incongruous pedigrees. This indicates that using stringent constraints for subsetting variables may result in greater vulnerability to outliers. In simulations we have carried out to assess the performance of SSCA (results not shown) our method demonstrates better performance compared to standard CCA based on full SVD in terms of test sample correlation (EP, unpublished data). Thus, SCCA may potentially provide a more robust solution. Additional empirical studies using more homogeneous sets of pedigrees and larger sample size would be useful.

## Competing interests

The author(s) declare that they have no competing interests.
